# ﻿A survey of the genus *Himalaphantes* Tanasevitch, 1992 (Araneae, Linyphiidae) with description of three new species from Yunnan, China

**DOI:** 10.3897/zookeys.1123.86261

**Published:** 2022-10-03

**Authors:** Meng-ting Zhang, Ping Liu, Muhammad Irfan, Xian-jin Peng

**Affiliations:** 1 College of Life Sciences, Hunan Normal University, Changsha, Hunan 410081, China Hunan Normal University Changsha China; 2 Key Laboratory of Eco-environments in Three Gorges Reservoir Region (Ministry of Education), School of Life Sciences, Southwest University, Chongqing 400715, China Southwest University Chongqing China

**Keywords:** Gaoligong Mountains, morphology, Southwest China, taxonomy

## Abstract

Three new species of *Himalaphantes* Tanasevitch, 1992 from Yunnan province, China, are described: *H.arcuatus***sp. nov.** (♀), *H.lingulatus***sp. nov.** (♂♀), and *H.uncatus***sp. nov.** (♂♀). The diagnosis of the genus is clarified, and extended detailed descriptions, photographs of somatic features and copulatory organs, and a distribution map are provided.

## ﻿Introduction

The genus *Himalaphantes* was erected by Tanasevitch (1992) to accommodate four ex-*Lepthyphantes* species: *Himalaphantesazumiensis* (Oi, 1979), *H.grandiculus* (Tanasevitch, 1987), *H.magnus* (Tanasevitch, 1987), and *H.martensi* (Thaler, 1987), which are distributed in China, India, Japan, Nepal, and Russia (WSC 2022). *Himalaphantesazumiensis* was reported from the Qinghai, Henan, Sichuan, and Hunan provinces of China (Zhu et al. 1986; Hu 2001; Zhu and Zhang 2011; Yin et al. 2012).

While examining specimens collected from the Gaoligong Mountains, Yunnan, three new species of the genus *Himalaphantes* were recognized and are described here. The genus diagnosis is clarified and extended due to the appearance of new congeners.

## ﻿Materials and methods

Specimens were stored in 75% ethanol. Epigynes were cleared in trypsin enzyme solution before examination and photography. Left male palps were used for description and color photographs. Specimens were examined and measured with a Leica M205C stereomicroscope. Photographs were taken using Kuy Nice E31SPM digital camera mounted on an Olympus BX53. Compound focus images were generated using Helicon Focus v. 7.6.1.0. A map was created using the online mapping software SimpleMappr (Shorthouse 2010) and then modified in Adobe Photoshop CS2. Leg chaetotaxy is given in the following order: (dorsal, proximal lateral, distal lateral, ventral). Leg measurements are given in the following order: total length (femur, patella + tibia, metatarsus, tarsus). All measurements are given in millimeters (mm). All type specimens treated in this study are deposited at the College of Life Sciences, Hunan Normal University, Changsha, China. The terminology used in the text and figures follows Tanasevitch (1992).

Abbreviations used in the text and figures are as follows: **ALE** = anterior lateral eyes; **AME** = anterior median eyes; **AME–ALE** = distance between AME and ALE; **AME–AME** = distance between AME; **apo** = anterior pocket of paracymbium; **appo** = apical pocket of paracymbium; **DSA** = distal suprategular apophysis; **E** = embolus; **EP** = embolus proper; **LC** = lamella characteristica; **LP** = lateral pocket; **fg** = Fickert’s gland; **MM** = median membrane; **PC** = paracymbium; **PCA** = proximal cymbial apophysis; **PLE** = posterior lateral eyes; **PME** = posterior median eyes; **PME–PLE** = distance between PME and PLE; **PME–PME** = distance between PME; **PMP** = posterior median plate; **ppo** = posterior pocket of paracymbium; **PS** = proscapus; **R** = radix; **S** = spermatheca; **ST** = subtegulum; **St** = stretcher; **T** = tegulum; **TA** = terminal apophysis; **TH** = thumb.

## ﻿Taxonomy

### ﻿Family Linyphiidae Blackwall, 1859

#### ﻿Genus Himalaphantes

Taxon classificationAnimaliaAraneaeLinyphiidae

Tanasevitch, 1992

8D62C6F0-C02A-523C-BE33-B160A5B483C1

##### Diagnosis.

**Figure 1.**
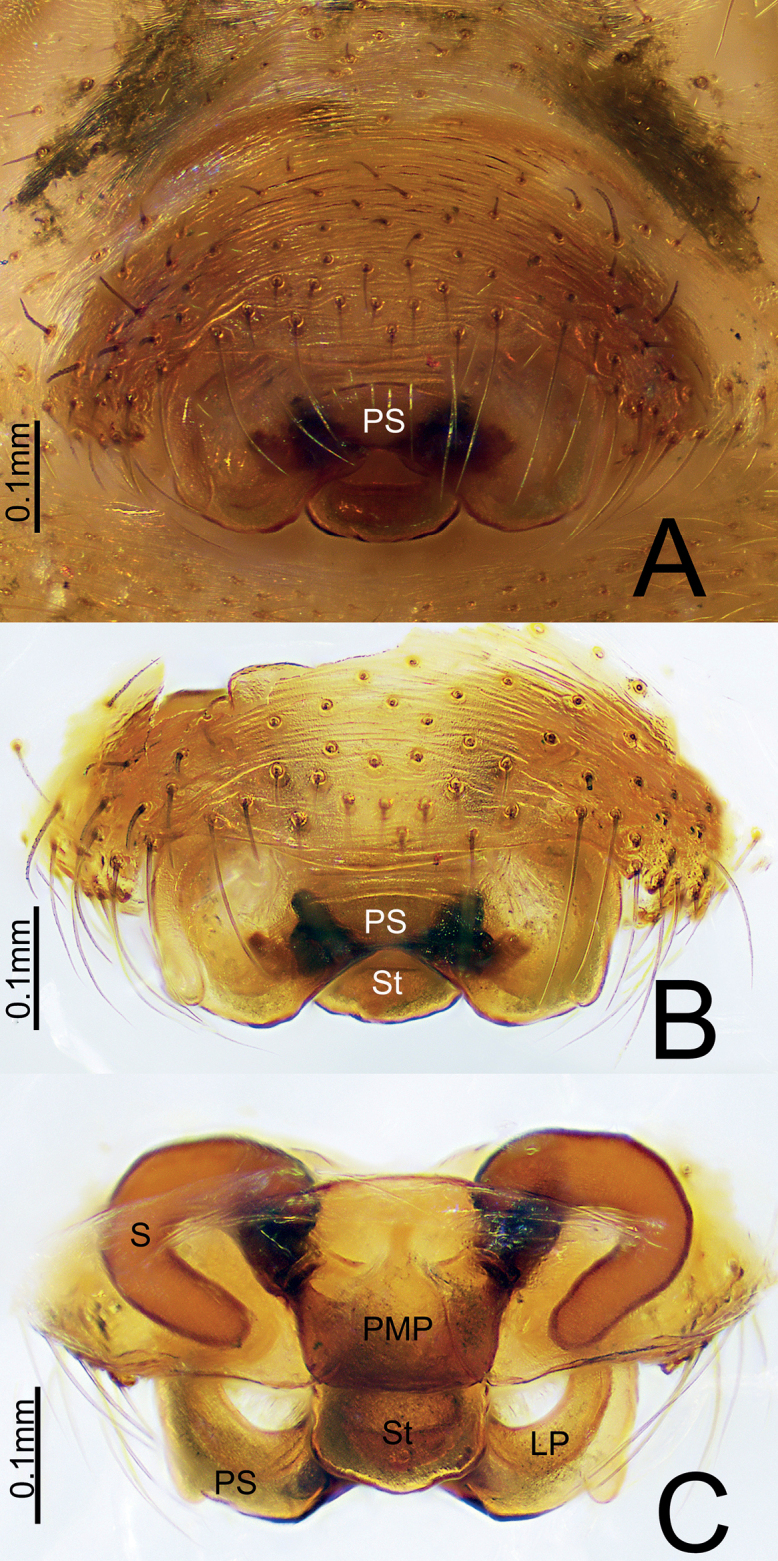
*Himalaphantesarcuatus* sp. nov., holotype ♀ **A, B** epigyne, ventral view **C** epigyne, dorsal view.

*Himalaphantes* is closely related to *Herbiphantes* Tanasevitch, 1992 in having the similar long legs, male palp tibia, modified male chelicerae and similar morphology of embolic division in palp (Tanasevitch 1992: fig. 1b, d, f), but it can be distinguished by the following features: posterior pocket of paracymbium with well-developed projection with blunt (Zhu and Zhang 2011: fig. 80D, E) to bifurcated end (Figs 4B, D, 7B, D), whereas posterior pocket of paracymbium absent in *Herbiphantes* (Tanasevitch 1992: fig. 1a, c, e). Well-developed proximal cymbial apophysis in *Himalaphantes* species (Figs 4A–C, 7A–C; Tanasevitch 1987: figs 1–3), whereas absent in *Herbiphantes* (Irfan and Peng 2019: figs 4A, B, D, 5A, B; Tanasevitch 1992: fig. 1a, c, e). Female epigyne can be distinguished from *Herbiphantes* species by the proscape small/enlarged with posterior margin smooth and/or posterior margin with small protuberance laterally in *Himalaphantes* species (Figs 1A, B, 5A, B, 8A, B; Tanasevitch 1992: figs 4–9), whereas posterior margin of proscape lacks any of small protuberance laterally in *Herbiphantes* (Irfan and Peng 2019: figs 6A–C, 7A, B; Tanasevitch 1992: fig. 2a–h); stretcher present in *Himalaphantes* species (Figs 1A–C, 5A–C, 8A–C; Tanasevitch 1992: figs. 4–9), whereas stretcher absent in *Herbiphantes* (Irfan and Peng 2019: figs 6A–C, 7A, B; Tanasevitch 1992: fig. 2a–h); posterior median plate relatively reduced and unmodified in *Himalaphantes* species (Figs 1C, 5C, 8C; Tanasevitch 1992: fig. 3e), whereas enlarged and modified in *Herbiphantes* (Irfan and Peng 2019: figs 6A–C, 7A, B; Tanasevitch 1992: fig. 2b, f, i).

##### Composition.

By addition of three new congeners, the genus *Himalaphantes* now comprises of seven species: *H.arcuatus* sp. nov. ♀, from China; *H.azumiensis* from Russia, Japan, and China; *H.grandiculus* from Nepal; *H.lingulatus* sp. nov. ♂♀, from China; *H.magnus* from Nepal; *H.martensi* (Thaler, 1987) from India and Nepal; and *H.uncatus* sp. nov. ♂♀, from China.

##### Distribution.

China, India, Japan, Nepal and Russia.

#### ﻿ Himalaphantes arcuatus sp. nov.

Taxon classificationAnimaliaAraneaeLinyphiidae

9F15A028-CF9E-5423-A55C-778888589A68

https://zoobank.org/F23021F9-AAA4-4CFF-974F-07AD6B200EEB

Figs 1 , 2 , 10

##### Type material.

***Holotype*** ♀: **China, Yunnan Province**: Longling County, Xiaoheishan Village, 24.5035°N, 98.4571°E, 2106 m, 29.X.2003, Guo Tang leg. (031029). ***Paratypes***: 17♀♀, same data as holotype (031029).

##### Etymology.

The specific epithet is derived from the Latin adjective “*arcuata*” (arched), referring to the arched spermatheca.

##### Diagnosis.

**Figure 2.**
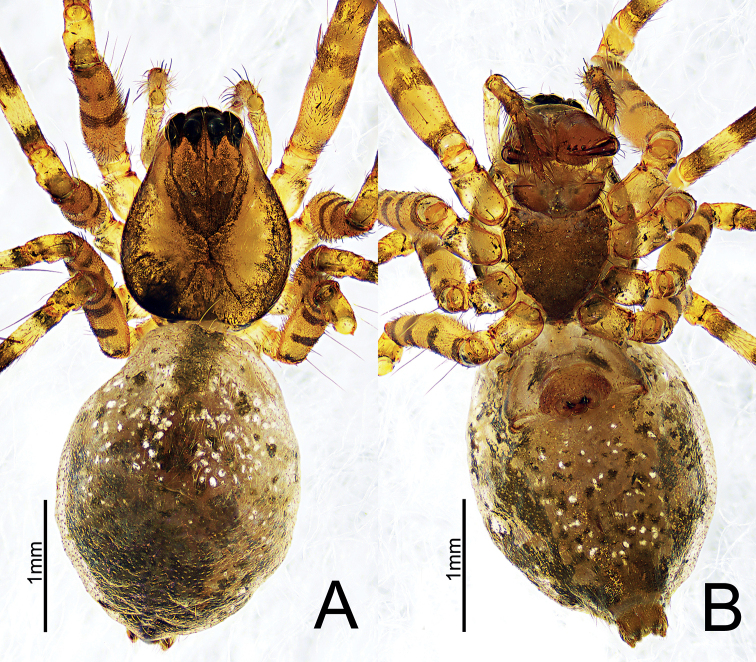
*Himalaphantesarcuatus* sp. nov., holotype ♀ **A** habitus, dorsal view **B** habitus, ventral view.

**Figure 3.**
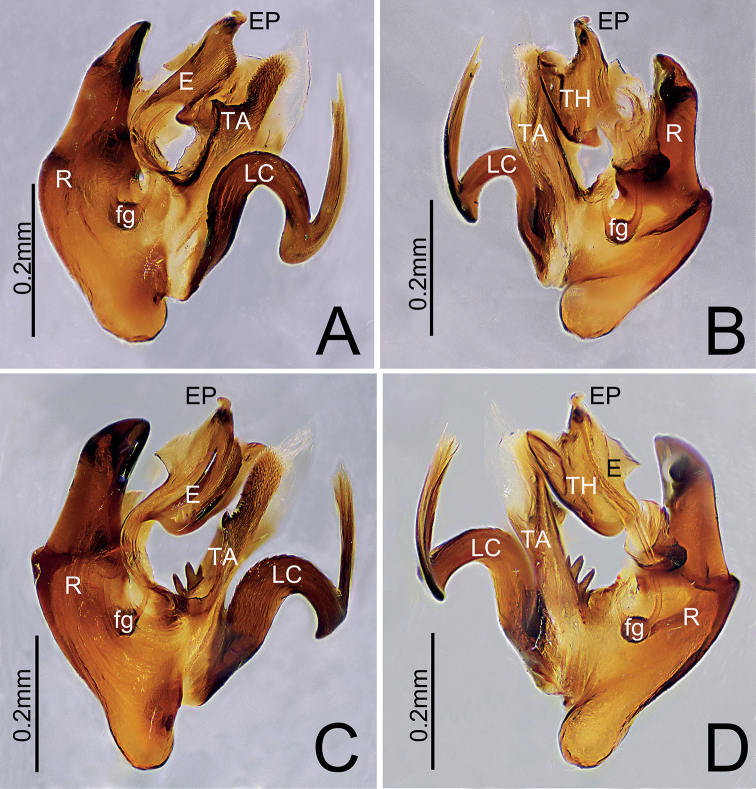
**A, B***Himalaphanteslingulatus* sp. nov., holotype ♂ **C, D***Himalaphantesuncatus* sp. nov., holotype ♂ **A, C** embolic division, prolateral view **B, D** embolus, retrolateral view.

This new species resembles *Himalaphantesuncatus* sp. nov. (Fig. 8), but can be distinguished by: (1) stretcher wider than long, with rounded end in *H.arcuatus* sp. nov. (Fig. 1C), whereas as wide as long, posterior margin with depression medially in *H.uncatus* sp. nov. (Fig. 8C); (2) spermathecae C-shaped in *H.arcuatus* sp. nov. (Fig. 1C), whereas sinuous in *H.uncatus* sp. nov. (Fig. 8C); (3) chelicerae with four retromarginal teeth in *H.arcuatus* sp. nov., whereas with five retromarginal teeth in *H.uncatus* sp. nov.

##### Description.

**Female** (holotype) (Fig. 2A, B). Total length 3.60. Carapace 1.12 long, 1.16 wide, yellow, sides brown, cephalic region slightly elevated, fovea, cervical and radial grooves distinct; clypeus 0.19 high. Sternum scutiform, brown. Endites brown, distal end broad with scopulae. Labium brown, wider than long. Chelicerae brown, with three promarginal and four retromarginal teeth. Eye sizes and interdistances: AME: 0.09, ALE: 0.11, PME: 0.07, PLE: 0.09, AME–AME: 0.06, AME–ALE: 0.08, PME–PME: 0.04, PME–PLE: 0.06, ALE–PLE: 0.03. Legs yellow with dark annuli. Spines: femur I–II: 1-1-0-0, III–IV: 0-0-0-0; tibia I–II: 2-2-2-2, III: 2-1-2-1, IV 2-2-2-1; metatarsus I, IV: 1-1-1-0, II–III: 1-1-1-1. Leg measurements: I, 8.01 (2.17, 2.77, 2.39, 0.68); II, 7.07 (1.75, 2.19, 1.97, 1.16); III, 4.98 (1.28, 1.46, 1.47, 0.77); IV, 6.65 (1.21, 2.07, 1.81, 1.56); leg formula 1243. Abdomen 2.41 long, 1.73 wide, oval, dorsum greyish yellow, with a dark longitudinal band and light spots dispersed anteriorly, irregular dark markings posteriorly; ventrum grayish yellow, with irregular dark or light spots.

Epigyne (Fig. 1A–C). Wider than long, proscapus wider than long, posterior margin with a deep depression medially, each side with one small protuberance; stretcher longer than wide, with rounded end. Posterior median plate trapezoid, covering most of the stretcher. Copulatory opening present in the middle of proscapus posteriorly. Copulatory ducts short, slightly curved. Spermathecae C-shaped.

**Male.** Unknown.

##### Distribution.

Known only from the type locality (Fig. 10).

#### ﻿ Himalaphantes lingulatus sp. nov.

Taxon classificationAnimaliaAraneaeLinyphiidae

62FC0992-E6D3-5C05-920D-5FA615BA536C

https://zoobank.org/9D6376F1-6F50-4DD1-AC61-A2A7262A55A1

**Figure 4.**
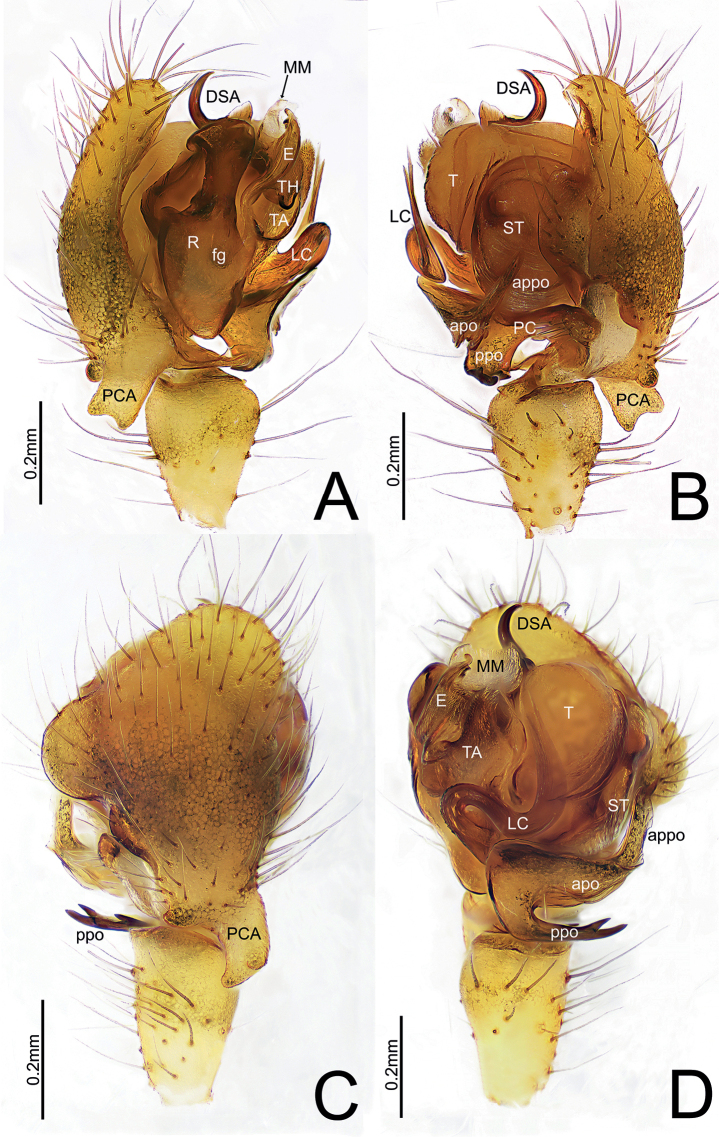
*Himalaphanteslingulatus* sp. nov., holotype ♂ **A** palp, prolateral view **B** palp, retrolateral view **C** palp, dorsal view **D** palp, ventral view.

**Figure 5.**
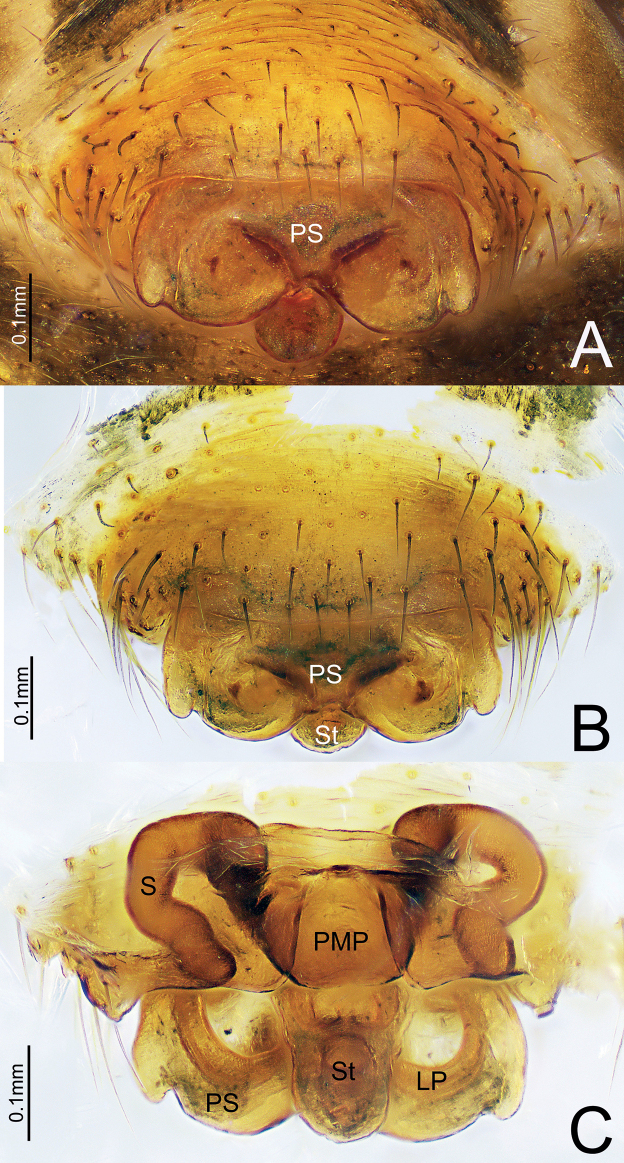
*Himalaphanteslingulatus* sp. nov., paratype ♀ **A, B** epigyne, ventral view **C** epigyne, dorsal view.

**Figure 6.**
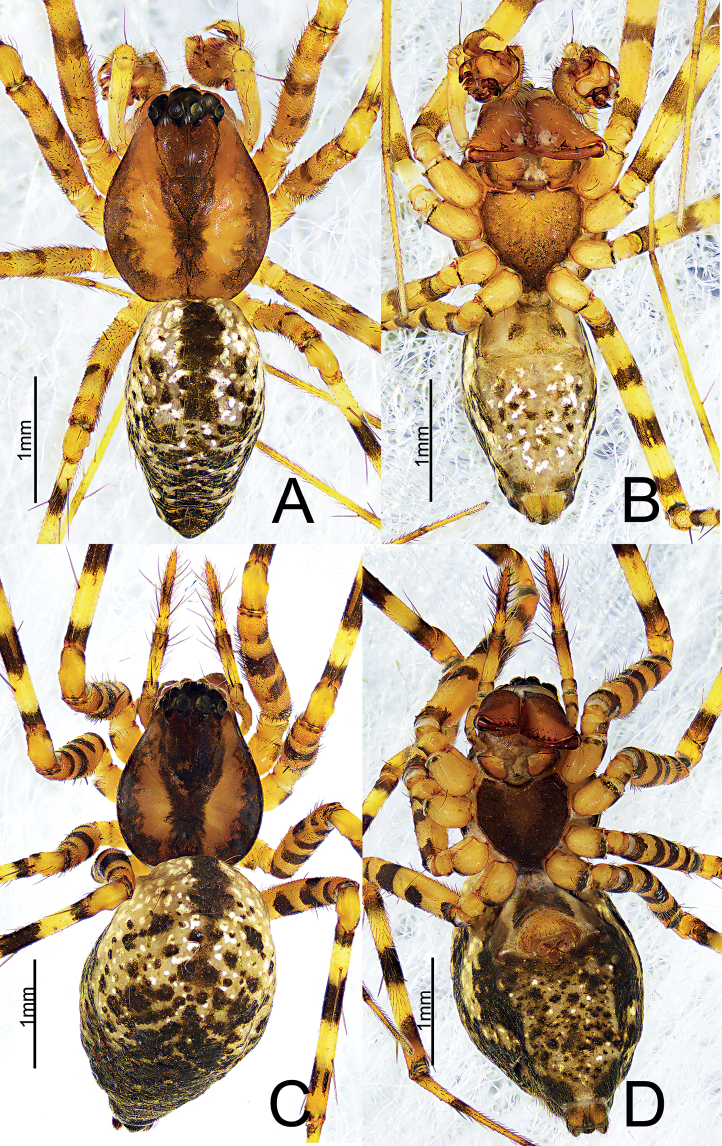
*Himalaphanteslingulatus* sp. nov., holotype ♂ and paratype ♀ **A** habitus, dorsal view **B** habitus, ventral view **C** habitus, dorsal view **D** habitus, ventral view.

Figs 3 , 4 , 5 , 6 , 10

##### Type material.

***Holotype*** ♂: **China, Yunnan Province**: Baoshan City, Yakou Village, 24.4372°N, 98.4605°E, 2186 m, 31.X.2003, Guo Tang leg. (Tang031031). ***Paratypes***: 1♂22♀♀, same data as holotype (Tang031031).

##### Etymology.

The specific epithet is derived from the Latin adjective “*lingulate*” (tongue-shaped), referring to the tongue-shaped stretcher.

##### Diagnosis.

This new species resembles *H.grandiculus* (Tanasevitch 1987: figs 2, 4–7, 10–12, 1992: fig. 3a–c) but can be distinguished by the following characters: (1) distal end of proximal cymbial apophysis depression medially in *H.lingulatus* sp. nov. (Fig. 4A), whereas rounded in *H.grandiculus* (Tanasevitch 1992: fig. 11); (2) distal branch of paracymbium near cymbiform in ventro-retrolateral view and with three teeth at midlength in *H.lingulatus* sp. nov. (Fig. 4B), whereas near flag-shaped and with one tooth in *H.grandiculus* (Tanasevitch 1987: fig. 2); (3) distal end of embolus blunt and curved in *H.lingulatus* sp. nov. (Fig. 3A), whereas pointed and straight in *H.grandiculus* (Tanasevitch 1992: fig. 3a); (4) stretcher about one-quarter width of scapus in *H.lingulatus* sp. nov. (Fig. 5A), whereas about one-fifth width of scapus in *H.grandiculus* (Tanasevitch 1987: fig. 4); (5) shape of anterior and lateral margins of epigyne arched in *H.lingulatus* sp. nov. (Fig. 5A); whereas varies from rounded to angular in *H.grandiculus* (Tanasevitch 1992: figs 4, 6)

##### Description.

**Male** (holotype) (Fig. 6A, B). Total length 3.59. Carapace 1.41 long, 1.18 wide, yellowish brown, with a brown longitudinal band medially, lateral sides brown, cephalic region slightly elevated, fovea, cervical and radial grooves distinct; clypeus 0.20 high. Sternum scutiform, yellowish brown with dark margin. Endites yellow, distal end broad with scopulae. Labium wider than long, yellowish brown. Chelicerae yellowish brown, with three promarginal and four retromarginal teeth. Eye sizes and interdistances: AME: 0.09, ALE: 0.11, PME: 0.12, PLE: 0.09, AME–AME: 0.04, AME–ALE: 0.05, PME–PME: 0.06, PME–PLE: 0.08, ALE–PLE: 0.06. Legs yellow with dark annuli. Spines: femur I: 0-1-0-0, II–IV: 0-0-0-0; tibia I: 2-2-1-2, II: 2-0-1-2 III–IV 2-0-1-1; metatarsus I–IV: 1-1-1-0. Leg measurements: I, 11.5 (2.68, 3.66, 3.54, 1.62); II, 8.89 (2.46, 2.61, 2.60, 1.22); III, 7.43 (1.27, 2.49, 2.41, 1.26); IV, 7.42 (2.60, 1.60, 2.27, 0.95); leg formula 1234. Abdomen 1.83 long, 0.88 wide, oval, yellow, dorsum with a dark longitudinal band and light spots dispersed anteriorly, dark herringbones posteriorly; ventral yellow, with lots of irregular dark or light patches.

Palp (Figs 3A, B, 4A–D). Tibia longer than wide. Cymbium longer than wide, median part of prolateral side bulged, proximal cymbial apophysis columnar, distal end as wide as base, with a shallow depression medially. Paracymbium sclerotized, apical pocket near cymbiform in in ventro-retrolateral and prolateral view, anterior pocket unmodified with smooth margin, posterior pocket with three teeth. Distal suprategular apophysis C-shaped, with pointed tip in retrolateral view. Radix longer than wide. Fickert’s gland present within radix. Lamella characteristically S-shaped, with V-shaped tip. Median membrane wider than long. Terminal apophysis proximally strongly sclerotized and distal end relatively membranous. Embolus broad and extending upwards, with curved and blunt end.

**Female** (one paratype of Tang031031) (Fig. 6C, D). Total length 3.99. Carapace 1.51 long, 1.22 wide, cervical and radial grooves indistinct; clypeus 0.25 high. Chelicerae with four promarginal and five retromarginal teeth. Eye sizes and interdistances: AME: 0.11, ALE: 0.13, PME: 0.12, PLE: 0.10, AME–AME: 0.03, AME–ALE: 0.04, PME–PME: 0.05, PME–PLE: 0.05, ALE–PLE: 0.02. Spines: femur I: 1-1-0-0, II–IV: 1-0-0-0; tibia I: 2-2-2-2, II: 2-1-2-2, III: 2-2-1-1, IV: 2-2-2-1; metatarsus I–IV: 1-1-1-0. Leg measurements: I, 6.84 (1.79, 2.78, 1.28, 0.99); II, 6.61 (1.92, 2.32, 1.32, 1.05); III, 5.43 (1.64, 1.57, 1.42, 0.80); IV, 6.01 (2.06, 2.10, 1.08, 0.77); leg formula 1243. Abdomen 2.42 long, 1.67 wide. Patterns same as in male, but darkly colored.

Epigyne (Fig. 5A–C). Wider than long, proscapus wider than long, posterior margin with a deep depression medially, each side with a small protuberance; stretcher much longer than wide in dorsal view, tongue-shaped with rounded end. Posterior median plate somewhat oval. Copulatory opening present in lateral pockets at the middle of proscapus posteriorly. Copulatory ducts short, slightly curved. Spermathecae tubular and sinuous.

##### Distribution.

Known only from the type locality (Fig. 10).

#### ﻿ Himalaphantes uncatus sp. nov.

Taxon classificationAnimaliaAraneaeLinyphiidae

C59666C5-19BF-5B40-9E9D-B1AF96FAD624

https://zoobank.org/F5C7648F-B52F-4684-AAD7-F53C4F979A0C

**Figure 7.**
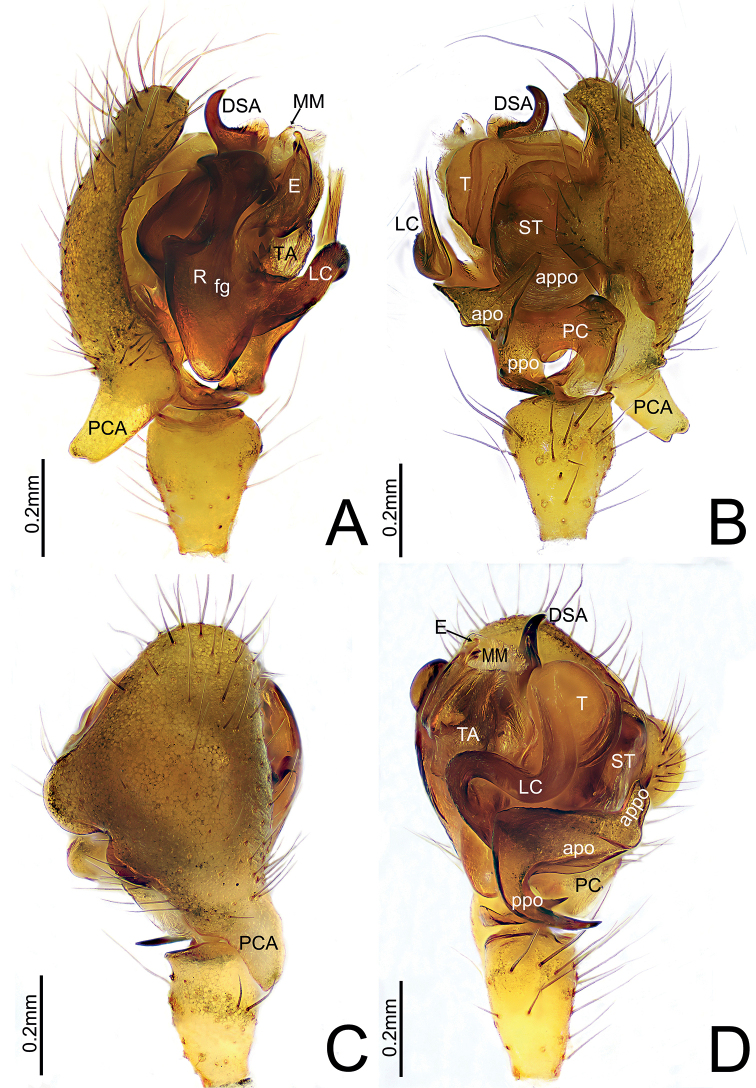
*Himalaphantesuncatus* sp. nov., holotype ♂ **A** palp, prolateral view **B** palp, retrolateral view **C** palp, dorsal view **D** palp, ventral view.

**Figure 8.**
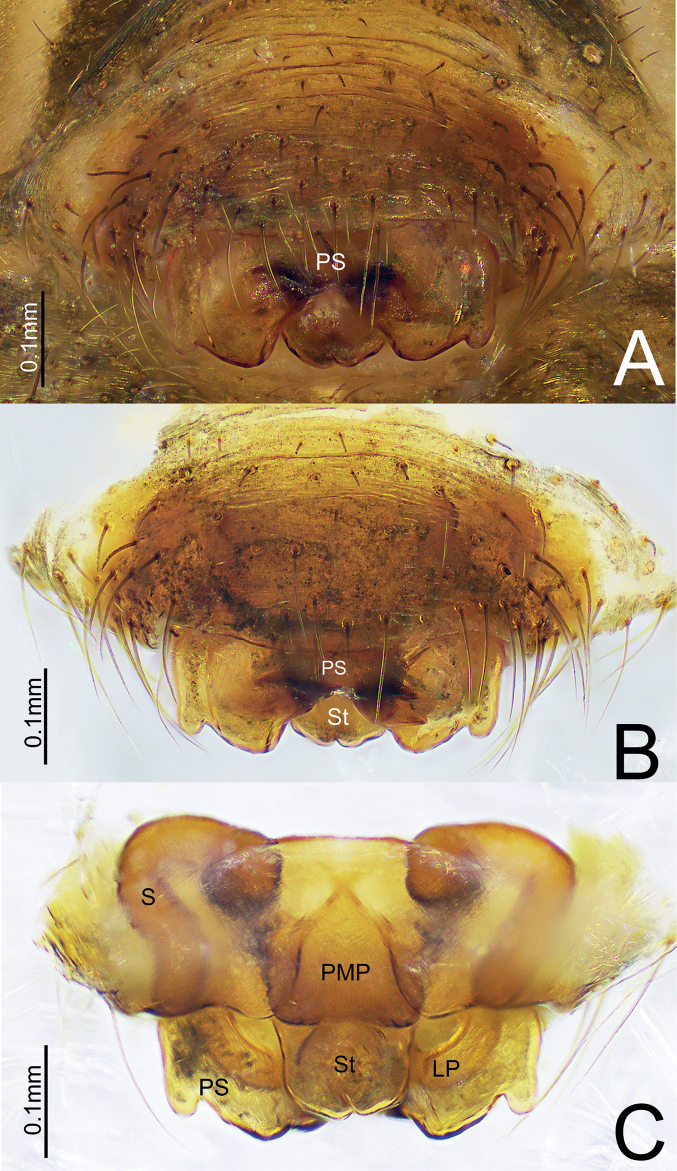
*Himalaphantesuncatus* sp. nov., paratype ♀ **A, B** epigyne, ventral view **C** epigyne, dorsal view.

**Figure 9.**
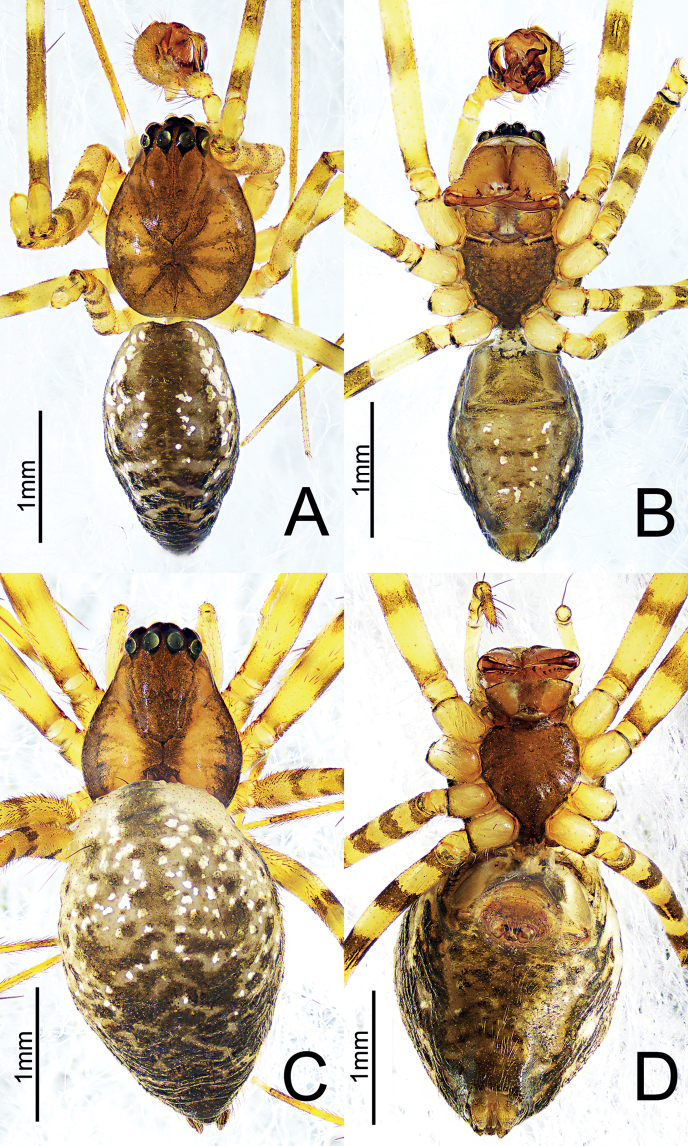
*Himalaphantesuncatus* sp. nov., holotype ♂ and paratype ♀ **A** habitus, dorsal view **B** habitus, ventral view **C** habitus, dorsal view **D** habitus, ventral view.

**Figure 10.**
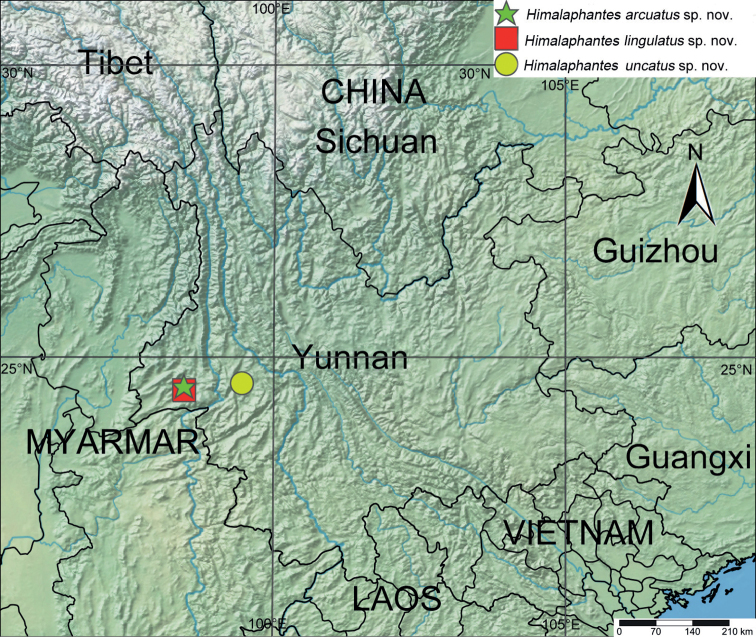
Type localities of *Himalaphantesarcuatus* sp. nov., *Himalaphanteslingulatus* sp. nov. and *Himalaphantesuncatus* sp. nov.

Figs 3 , 7 , 8 , 9 , 10

##### Type material.

***Holotype*** ♂: **China, Yunnan Province**: Tengchong County, Dahaoping Village, km 41–46 on the road from Bawan to Tengchong, 24.5563°N, 99.4516°E, 2416 m, 18.X.2003, Guo Tang leg. (Tang031018). Paratypes: 1♂18♀♀, same data as holotype (Tang031018).

##### Etymology.

The specific epithet is derived from the Latin adjective “*uncatus*” (hook-shaped), referring to the hook-shaped distal suprategular apophysis.

##### Diagnosis.

The new species resembles *Himalaphanteslingulatus* sp. nov. (Figs 3–6) but can be distinguished by the following characters: (1) proximal cymbial apophysis narrowing posteriorly in prolateral view in *H.uncatus* sp. nov. (Fig. 7A), whereas somewhat rectangular in *H.lingulatus* sp. nov. (Fig. 4A); (2) anterior pocket of paracymbium triangular in retrolateral view in *H.uncatus* sp. nov. (Fig. 7B), whereas somewhat cymbiform in *H.lingulatus* sp. nov. (Fig. 4B); (3) posterior margin of proscapus with a deep inverted V-shaped depression in *H.uncatus* sp. nov. (Fig. 8A–C), whereas with a transverse arc-shaped depression in *H.lingulatus* sp. nov. (Fig. 5A–C); (4) stretcher almost as long as wide, posterior margin slightly depressed medially in dorsal view in *H.uncatus* sp. nov. (Fig. 8B), whereas much longer than wide, with rounded end in *H.lingulatus* sp. nov. (Fig. 5B).

##### Description.

Male (holotype) (Fig. 9A, B). Total length 3.13. Carapace 1.36 long,1.06 wide, yellowish brown, with brown lateral side, cephalic region slightly elevated, with brown lines from posterior lateral eyes to fovea, fovea, cervical and radial grooves distinct; clypeus 0.17 high. Sternum scutiform, brown. Endites yellowish brown, distal end broad with scopulae. Labium wider than long, brown. Chelicerae yellowish brown, with three promarginal and five retromarginal teeth. Eye sizes and interdistances: AME: 0.08, ALE: 0.10, PME: 0.12, PLE:0.11, AME–AME: 0.03, AME–ALE: 0.05, PME–PME: 0.04, PME–PLE: 0.05, ALE–PLE: 0.06. Legs yellow with dark annuli. Spines: femur I–IV: 1-0-0-0; tibia I–II: 2-1-1-2, III: 2-1-2-1, IV: 2-1-1-1; metatarsus I–IV: 1-1-1-0. Leg measurements: I, 10.88 (2.96, 3.28, 3.36,1.28); II, 8.13 (2.45, 2.13, 2.33, 1.22); III, 5.77 (1.42, 1.57, 1.71, 1.07); IV, 6.81 (1.88, 1.85, 2.12, 0.96); leg formula 1243. Abdomen 1.69 long, 0.95 wide, oval, dorsum greyish yellow, with three or four dark herringbones posteriorly and irregular white patches at median and lateral sides; ventral greyish yellow with a few white patches medially.

Palp (Figs 3C, D, 7A–D). Tibia longer than wide. Cymbium longer than wide, median part of retrolateral margin bulged, proximal cymbial apophysis almost cylindric, distal end narrow than base, with a shallow depression medially. Paracymbium sclerotized, apical pocket finger-shaped with blunt end, anterior pocket somewhat triangular in retrolateral view, posterior pocket with three teeth at mid length. Distal suprategular apophysis hook-shaped, with pointed tip in retrolateral view. Radix much longer than wide. Fickert’s gland present within radix. Lamella characteristically S-shaped in ventral view. Median membrane wider than long. Terminal apophysis with four teeth at the base, proximally strongly sclerotized and distal end relatively membranous. Embolus broad and extending upwards, with curved and blunt tip, thumb well-developed.

**Female** (one paratype of Tang031018) (Fig. 9C, D). Total length 3.63. Carapace 1.09 long, 1.11 wide, cervical and radial grooves indistinct; clypeus 0.14 high. Chelicerae with three promarginal and five retromarginal teeth. Eye sizes and interdistances: AME: 0.09, ALE: 0.10, PME: 0.11, PLE: 0.12, AME–AME: 0.03, AME–ALE: 0.07, PME–PME: 0.05, PME–PLE: 0.04, ALE–PLE: 0.01. Spines: femur I: 0-1-0-0, II–IV: 0-0-0-0; tibia I: 2-2-1-3, II–IV: 2-2-2-1; metatarsus I–II: 1-1-1-0, III–IV: 1-1-1-1. Leg measurements: I, 6.36 (2.22, 1.55, 1.66, 0.93); II, 9.24 (1.74, 2.80, 3.20, 1.50); III, 4.61 (1.44, 1.39, 0.97, 0.81); IV, 5.31 (1.70, 1.40, 1.44, 0.77); leg formula 2143. Abdomen 2.42 long, 1.66 wide. Color and patterns same as in male.

Epigyne (Fig. 8A–C). Wider than long, proscapus wider than long, posterior margin with a deep depression medially, each side with a small protuberance; stretcher almost as long as wide, posterior margin slightly depressed medially. Posterior median plate somewhat rectangular. Copulatory opening present in the middle of scapus posteriorly. Copulatory ducts short, slightly curved. Spermathecae tubular, sinuous.

##### Distribution.

Known only from the type locality (Fig. 10).

## Supplementary Material

XML Treatment for Himalaphantes

XML Treatment for Himalaphantes arcuatus

XML Treatment for Himalaphantes lingulatus

XML Treatment for Himalaphantes uncatus
